# Supplementary material for: Are carbon emissions trading and green financial instruments synergistic? -Comprehensive quantitative research based on content analysis
Source: PLoS One. 2024 Mar 7;19(3):e0298601. doi: 10.1371/journal.pone.0298601 (PMC10919601; doi:10.1371/journal.pone.0298601)
Supplement: S1 Data — (ZIP) [file pone.0298601.s001.zip › Data/地级市Z_F工作报告.zip!/║╙─╧/╔╠╟≡╩╨/└┤╫╘╣1⁄2╓┌║┼í╛┬φ┐╦╩2╛▌═°í┐_2018.docx]

各位代表：

现在，我代表市人民政府，向大会报告工作，请予审议，并请各位政协委员和其他列席人士提出意见。

一、2017年工作回顾

2017年，在省委、省政府和市委的正确领导下，全市上下认真学习贯彻党的十九大精神，以习近平新时代中国特色社会主义思想为指导，坚持稳中求进工作总基调，以供给侧结构性改革为主线，积极对接“一带一路”和“三区一群”战略，紧紧围绕“六区一中心”工作思路和“4233”奋斗目标，认真实施九大跨越发展行动计划和2017年行动方案，以重大项目建设为抓手，统筹推进各项重点工作，较好地完成了市五届人大一次会议确定的各项目标任务。

(一)经济发展在扩量提质中不断优化。初步预计，全市生产总值完成2220亿元，增长8.5%左右。固定资产投资2230亿元，增长12%。一般公共预算收入128.8亿元，增长15.2%。社会消费品零售总额1030亿元，增长12.2%。城乡居民人均可支配收入16530元，增长10%，其中城镇居民人均可支配收入27490元，增长9%，增速居全省第1位；农民人均可支配收入10450元，增长8.8%，增速居全省第3位。主要经济指标均高于全国、全省平均水平，实现了“三个高于”的目标。产业结构持续优化，预计三次产业结构比为19.2∶40.6∶40.2，第三产业占GDP比重较上年提高1个百分点左右。新兴动能加快成长，新增国家级高新技术产业化基地1家，总数居全省第1位；新增国家级科技企业孵化器1家，总数居全省第3位；新增高新技术企业26家，总数居全省第5位。高新技术产业、六大高成长性制造业增加值分别增长13.1%、13.2%，占全市规模以上工业增加值的比重较上年分别提高1.5个、2.3个百分点。金振源电子税收突破1亿元，示范区财政收入突破10亿元，科迪集团产值突破100亿元。

(二)产业发展在结构调整中转型升级。以供给侧结构性改革为主线，落实“三去一降一补”措施，坚决淘汰落后产能，5家“地条钢”制售企业全部依法取缔，25家扭亏无望企业和4家“僵尸企业”完成清理处置。通过大力调整产业结构，工业用电量由上半年的负增长，逐步填平回升，并保持稳定增长；工业税收同比增长25.3%。招商引资和项目建设取得新突破，全市新上3亿元以上项目59个，实际利用外资3.4亿美元，实际利用省外资金625亿元，利用省外资金总量居全省第3位。智能制造取得新突破，实施设备换芯、生产换线、机器换人，83家企业完成机器换人，6家企业被评为河南省智能工厂、智能车间，7家企业入选河南省智能制造试点；新落地的神火集团20万吨双零铝箔、永安纺织100万锭智能化纤纺、华晶金刚石等重大工业项目，符合智能化、信息化、绿色化生产要求，实现了工业总量提升和结构优化。现代服务业发展实现新突破，第三产业增加值同比增长10.5%，稳居全省前列。建成电子商务产业园区9个，阿里巴巴(商丘)产业带入驻企业1437家，综合排名居全国第12位，较前年提升了5个位次。快递业呈快速增长态势，业务量和业务收入分别增长56.0%、39.8%，总量分别居全省第3位、第4位。保税物流中心封关运营。金融业发展态势良好，平顶山银行、恒大寿险等7家金融机构落地，金融机构聚集度居全省前列。农业供给侧结构性改革取得新突破，建设高标准粮田60万亩，全年粮食总产136.2亿斤，居全省第3位，调整特色农业种植面积400万亩，调减玉米种植面积86.8万亩。柘城辣椒获国家地理标志产品保护，夏邑西瓜获批国家地理标志性农产品。流转土地225万亩，超过全市耕地总面积的四分之一。发展新型农业经营主体2959家，农业现代化程度和综合效益显著提升。

(三)脱贫攻坚在精准施策中扎实推进。紧紧围绕“6633”晋位争优目标，根据脱贫攻坚不同阶段、不同特点，采取不同措施，及时解决工作中的重点难点问题。按照新的标准，精准识别、精准建卡、精准立档，集中3个月时间，组织3万多名干部，忘我工作，吃住在村，走访79万户，精准建立了贫困档案，错评率、错退率均低于2%，为精准施策、精准脱贫奠定了坚实基础。采取产业结构调整、养殖单元下乡、扶贫车间普及等扶贫措施，推动农业产业化龙头企业助力脱贫攻坚，科迪集团万头奶牛现代牧场和高老庄食品6个万头生猪扶贫养殖小区建成投产，拓宽了贫困户增收渠道。加强低保救助与扶贫政策有效衔接，将8.6万建档立卡贫困人口纳入低保范围。建立了全面覆盖建档立卡家庭学生各个学段的资助政策体系。实施贫困村“九大工程”项目2321个、贫困户“六改工程”15.8万户，贫困村基础设施、生产生活条件和村容村貌发生重大变化。统筹整合财政资金30.7亿元，发放小额贷款47.8亿元，建成扶贫车间1034个，建成光伏电站323座，完成9.72万贫困人口稳定脱贫，200个贫困村退出贫困序列。健康扶贫走在全省前列，全省健康扶贫工作现场会在睢县召开；夏邑县、虞城县在全国脱贫攻坚考核巡查中得到充分肯定。

(四)环境质量在攻坚克难中不断提升。坚定不移贯彻落实省委、省政府坚决打赢环境治理攻坚战的各项工作部署，进一步完善环境污染防治的各项措施，环境质量持续好转，置换了发展空间，实现了环境质量与经济发展同步提升。全面实行城区烟花爆竹禁燃禁放，秸秆禁烧实现了全市“零火点”，中心城区12月份实行机动车限行，广大群众积极配合，为“商丘蓝”做出了贡献。全市空气质量优良天数227天，较去年增加了36天，PM10、PM2.5分别下降10%、19.5%，其中PM2.5降幅居全省第2位。全省环境污染攻坚扬尘治理现场会在我市召开，道路保洁联合作业模式、渣土运输“两固定、四严格”措施、拆迁四步法等商丘模式、商丘经验在全省推广。及早动员部署，全面建立落实河长制，开展黑臭水体综合治理，实施局部打通、黑臭水体抽排、黑臭淤泥清理、排污口封堵、污水管网铺设、护坡治理等九大工程，清理长年积存的黑臭污泥220万立方米。2月份我市水质改善幅度居全国第9位，3月份居第6位，受到环保部表扬。中心城区9条内河水质大幅改善，基本消除黑臭水体。5个出境断面水质全部达标，达标率较上年提高33个百分点。

(五)城乡面貌在百城提质中明显改善。坚持规划引领，完善规划编制体系，商丘市城乡总体规划顺利通过专家评审，6个县全部完成城乡总体规划编制工作。强力推进中心城区基础设施建设。实施了189条道路建设，主干道基本实现了连通，外环物流快速通道正在加快建设。日月湖、古城、高铁站周边片区路网建设有序推进。商合杭高铁开始铺轨，高铁枢纽换乘中心基本建成。“三环九放射”市域快速通道5条道路开工建设。宁陵县荣获首批“四好农村路”全国示范县。着力推动“四率”提升。中电国际2×35万千瓦热电联产项目进展顺利，新增供热面积528万平方米，供热普及率提高18.8个百分点。实施天然气供应项目68个，保障了市区生产生活用气需要。启动新水厂建设、老旧管网改造和吴屯水库取水口上移工程，供水能力进一步提升。第八污水处理厂建成运行，新增污水处理能力10万吨。中心城区垃圾焚烧发电项目开工建设。实施公园、绿地项目128个，启动12个便民公园建设，新增绿化面积45.7万平方米，顺利通过国家园林城市复查。商丘历史文化名城保护规划通过专家评审，划定了古城保护的历史街区，明确了重点保护范围，对新认定的72处历史建筑和古树名木，实行挂牌保护，东南城湖清淤、城墙修复等工程有序推进，带动全域旅游加快发展，全年接待游客增长18.1%，旅游收入增长19.7%。百城建设提质工程成效显著，市县两级谋划项目1049个，完成投资313亿元。夏邑县天龙湖生态治理、民权县梦蝶会展中心二期、虞城县人民公园改扩建等项目取得重大进展，部分已经建成投入使用。全省百城建设提质工程现场会在我市召开，城市环境的变化增强了广大市民的认同感和凝聚力，提升了广大人民群众的幸福感和自豪感，受到了外来客商和广大游客的广泛赞誉，全市人民对商丘的发展更加充满信心。棚户区改造进展顺利，国家政策性融资到位200多亿元，全市实施棚户区改造11.6万套3142万平方米，置换出建设用地6万多亩，供应各类建设用地226宗1.2万亩，其中新建道路、公园、水系、输变电站等基础设施用地近6000亩，出让6000多亩，出让面积和收益分别增长69%和192%。盘活问题楼盘28个340万平方米，有力促进了房地产市场健康发展，稳定了市场预期。

(六)发展活力在深化改革中不断释放。破除不适应、不符合发展要求的体制机制，加快促进项目落地，激发民间投资活力，使体制更顺、机制更活、干劲更足。深化“放管服”改革。推进简政放权，取消行政许可、行政处罚等行政职权52项，是全省保留行政许可最少的省辖市之一。推进“双随机一公开”，推行“互联网+政务服务”，整合12345政务服务热线，优化了营商环境，提升了政务服务水平。推进商事制度改革，实现了“三十五证合一”和证照全程电子化，市本级取消各类证明事项216项，市行政服务中心被确定为国家级服务业标准化示范单位。全市新增市场主体6.98万户，总量达到33万户，同比增加15.79%。稳步推进财税金融和投融资体制改革。落实营改增政策，为企业减税9.5亿元。大力推进政府和社会资本合作，强化财政承受能力论证，从严筛选项目，全市共落地PPP打捆项目17个，总投资333亿元。发起组建了7支基金，总规模280多亿元，截至目前已到位资金96亿元，开创了多项地市级基金全省第一，有力支持了我市城市基础设施建设和产业发展。推进市容环卫体制改革，按照政府主导、市场化运作的模式，组建政府平台公司，通过政府购买服务和第三方评估的方式拨付财政资金，迅速壮大和提升了市容环卫队伍的实力和机械化水平。全面推进农信社改革，完善了法人治理结构，置换了不良资产，优化了股权结构，支持了地方经济发展。扎实推进国有企业改革。在全省率先完成驻商省属以上企业“三供一业”剥离工作。神火集团关停合并子公司37个，压缩管理层级30%以上，焕发出新的生机和活力，在全省煤炭行业中率先扭亏为盈。坚定不移推进路桥集团、市政工程总公司等国企改制和盐业体制改革。路桥集团勇于改革，创新管理，经营逐步好转。积极推进户籍制度改革，全市户口迁移实现了迁入地一站式办理，农业转移人口落户城镇10万人。扎实推进农村改革，大力推进农村土地“三权”分置和农村集体资产改革，基本完成农村土地承包经营权确权登记颁证；启动供销社改革和国有林场改革，为农业供给侧结构性改革注入了强大动力。

(七)民生福祉在强化保障中持续增进。认真办好省市重点民生实事，继续加大投入力度，全市民生支出369亿元，占财政支出的79.7%。实施积极的就业政策，全市城镇新增就业6.6万人，完成省定目标任务的119%；农村劳动力转移就业7.3万人，是省定目标的159%。加快教育事业发展，超额完成省定“全面改薄”任务，新建、改扩建学校513所、农村寄宿制学校50所，89所公办幼儿园全部开工。市一高、一中新校区等项目进展顺利。商丘医专顺利完成搬迁。加强公共文化服务体系建设，深入实施“百戏进百乡”、农村公益电影放映等文化惠民工程，建成798个村级综合性文化服务中心，市文化艺术中心主体工程完工。加快医疗卫生事业发展，市第一人民医院儿科病房楼、市第四人民医院等项目建成投用，市中医院新院区、市传染病医院西院区等项目开工建设。城乡社会保障制度实现全覆盖，城乡居民养老、医疗、工伤和失业保险覆盖率均达95%以上，城镇居民医保财政补助标准人均提高30元，农村低保标准年人均提高190元，城市低保标准年人均提高360元，中心城区实现残疾人免费乘坐公交车。建成15家社区日间照料中心、600余所农村幸福院和一批社会养老机构，多层次、多样化的养老服务业格局正在形成。持续深化平安商丘建设，落实“三·五”基层工作日制度，深入开展基层矛盾纠纷排查化解活动，圆满完成党的十九大期间信访安全稳定工作任务，全市公众安全感指数达93.2%，居全省第3位。民权县荣获全国平安建设先进县。不断健全食品药品安全治理体系，确保了人民群众饮食用药安全。落实安全生产责任制，狠抓安全隐患排查治理，安全生产形势继续保持稳定。

深入开展精神文明创建，全面践行社会主义核心价值观，用“商丘好人”引领文明风尚。5人入选“中国好人榜”和全国道德模范候选人，“商丘好人”主题公园建成开放，永城市成功创建全国文明城市，柘城县获得创建全国文明城市提名城市，8家单位、学校、村镇荣获国家级文明称号，城乡文明程度明显提升。“七五”普法深入推进。持续加强国防教育、国防后备力量及人民防空建设，驻商解放军、武警和预备役部队为地方发展作出新贡献，军民融合深度发展，第八次蝉联省“双拥模范城”。对台、外事侨务、气象、史志、档案、统计、审计、烟草、机关事务、民族宗教和对口援疆等工作都取得了新成绩。

各位代表！这些成绩的取得，是省委、省政府和市委坚强领导的结果，是市人大、市政协有效监督、大力支持的结果，是全市人民团结拼搏的结果，也是社会各界共同努力的结果。在此，我代表市人民政府，向全市人民，向驻商解放军、武警官兵、公安民警，向各民主党派、工商联、人民团体和无党派人士，向各位人大代表、政协委员，向所有关心支持和参与商丘建设发展的同志们、朋友们，表示崇高的敬意和衷心的感谢！

在肯定成绩的同时，我们也清醒地认识到，当前经济社会发展中还存在一些困难和问题：一是底子薄，人均生产总值和财力与全国、全省平均水平相比还有较大差距。二是产业结构不合理，传统产业占比较高，科技创新能力不强，高端人才不足，高质量发展任务繁重。三是民生短板较多，文化、教育、医疗、养老等公共服务水平还需较大程度提升，脱贫攻坚任务艰巨。四是环境问题依然突出，大气污染防治、水污染治理、农村环境综合整治仍需下大力气。五是职能转变还不到位，一些政府工作部门还不能适应新时代新要求，缺乏应有的担当和开拓进取精神。对此，我们一定高度重视，认真加以解决。

二、2018年重点工作

2018年是贯彻党的十九大精神的开局之年，是改革开放40周年，是决胜全面建成小康社会、实施“十三五”规划承上启下的关键一年。做好2018年的工作，至关重要。党的十九大报告提出，中国特色社会主义进入新时代，我国经济已由高速增长阶段转向高质量发展阶段，正处在转变发展方式、优化经济结构、转换增长动力的攻关期。中国经济发展进入新时代，其重要标志就是高质量的发展。刚刚结束的中央经济工作会议，对今年经济工作进行了安排部署，突出主题就是高质量发展。结合商丘实际，落实好、推进好高质量发展，我们必须充分认识到，商丘当前的主要矛盾是发展不足和不充分的问题，不足主要表现为人均GDP和人均财力落后，不平衡是由于发展不足、不充分造成的，主要是城乡区域间发展的不平衡。破解这一矛盾，我们必须坚决贯彻习近平新时代中国特色社会主义经济思想，把推动高质量发展作为当前和今后一个时期确定发展思路、出台发展政策、推进发展措施的根本要求。推动高质量发展，必须把握好以下几个方面：一是必须深化对高质量发展的认识。深刻理解推动高质量发展的丰富内涵和重大意义，牢牢把握推动高质量发展的根本要求，切实转变发展理念，真正使创新成为第一动力、协调成为内在要求、绿色成为普遍形态、开放成为必由之路、共享成为根本目的。二是必须制定和完善推动高质量发展的措施。以九大跨越发展三年行动计划为基础，以高质量发展为根本要求，制定推动高质量发展十个专项工作方案和推动高质量发展的指标体系、政策体系、标准体系、统计体系、绩效评价和政绩考核体系。三是必须注重抓重点补短板强弱项推动高质量发展。紧紧围绕创新驱动、脱贫攻坚、乡村振兴、百城提质等重点工作，加快补齐医疗、教育、文化、环境、社区建设等方面的民生短板，真正使发展不断朝着更高质量、更有效率、更加公平、更可持续的方向前进。四是必须充分发挥优势推动高质量发展。立足商丘资源禀赋，引导资源要素优化配置和高效整合，通过交通枢纽优势彰显区位优势，将劳动力优势打造成人力资源优势，为高质量发展夯实基础。五是必须强化生态建设推动高质量发展。坚持标本兼治，集中治理环境突出问题，下决心淘汰退出落后产能，从严把好新上项目的生态环保关，加快构建以黄河故道为主脉，渠相通、湖相连、林相接的生态系统，走绿色发展之路。六是必须以新经济引领推动高质量发展。以新技术、新产业、新模式为核心，以知识、技术、信息、数据等新生产要素为支撑，聚焦互联网经济、数字经济、分享经济、绿色经济、智造经济等新业态，为高质量发展注入强大新动能。七是必须创新发展生态链推动高质量发展。实现高质量发展，核心是推动经济结构调整和产业转型升级，根本途径是走创新驱动发展的路子。坚持以孵化器、众创空间等为创新创业载体，加快人才公寓建设，出台吸引人才的专项激励政策，引入创新人才、高校毕业生在商丘创新创业。八是必须坚持以人民为中心的发展思想推动高质量发展。始终把人民群众对美好生活的向往作为我们的奋斗目标，不断强化公共产品供给，突出解决好人民群众关心的热点、难点问题，实现好、维护好、发展好最广大人民根本利益，使群众的获得感、幸福感更加充实、更有保障、更可持续。

2018年政府工作的总体要求是：深入贯彻党的十九大和中央、省委、市委经济工作会议精神，以习近平新时代中国特色社会主义思想为指导，持续落实习近平总书记调研指导河南工作时的重要讲话精神，坚持党对经济工作的领导，坚持稳中求进工作总基调，坚持新发展理念，紧扣我国社会主要矛盾变化，坚持高质量发展的根本方向，统筹推进“五位一体”总体布局、协调推进“四个全面”战略布局，坚持以供给侧结构性改革为主线，积极对接“三区一群”战略，着力发挥优势打好“四张牌”，按照市五次党代会提出的“四个总”工作要求，围绕“六区一中心”工作思路和“4233”奋斗目标，认真落实推动商丘高质量发展“一方案十专案”，统筹推进稳增长、促改革、调结构、惠民生、防风险各项工作，强力打好决胜全面建成小康社会三大攻坚战，强力推进国家区域中心城市建设，强力深化改革开放创新，促进经济社会持续健康发展，在决胜全面建成小康社会、开启新时代商丘全面建设社会主义现代化新征程中迈出坚实步伐。

全市经济社会发展的主要预期目标是：生产总值增长8.5%左右；一般公共预算收入增长8.5%；全社会固定资产投资增长11%；社会消费品零售总额增长11%；实际利用省外资金增长7%左右；居民消费价格涨幅3%左右；城乡居民收入高于经济增长速度；节能减排等约束性指标完成省定目标。实现上述目标，我们将按照高质量发展的要求，重点抓好以下十个方面的工作。

(一)坚决打好精准脱贫攻坚战。打赢脱贫攻坚战是全面建成小康社会的重要前提，是实现第一个百年奋斗目标的重要标志。坚持把脱贫攻坚放在统揽经济社会发展全局的战略地位，瞄准贫困群众精准帮扶，下足“绣花”功夫，拿出过硬办法，夯实脱贫责任，确保稳定脱贫。坚持精准方略。始终坚持精准扶贫精准脱贫，真正把扶持对象、项目安排、资金使用、措施到户、因村派人、脱贫成效“六个精准”落到实处，做到脱贫工作务实、脱贫过程扎实、脱贫结果真实。做好产业扶贫。加强1034个扶贫车间的建设和管理，引进适合本地发展的产业，实现扶贫车间由建设全覆盖到使用全覆盖。依托农业产业化龙头企业和农民专业合作社，支持乐为牧业等企业搞好养殖单元下乡，注重向边远贫困村倾斜。加快569个村级光伏发电项目建设进度，为贫困群众建立稳定、可持续的增收渠道，实现多种扶贫措施对所有贫困户全覆盖。做足土地文章，加快贫困村土地流转，把土地资本转化为贫困户增收的重要来源；加大金融小额贷款扶贫力度，把小额贴息贷款作为贫困户发展产业脱贫致富的重要支撑。抓好健康扶贫。实施9种大病专项救治，推进家庭医生签约服务，全面推行“先诊疗后付费”和“一站式”服务。完成贫困村标准化卫生室建设任务。推广睢县“防、服、减、保”健康扶贫政策和虞城县“四次报销”模式，破解因病致贫、因病返贫难题。统筹推进金融扶贫、教育扶贫、兜底扶贫等扶贫措施，打好行业扶贫组合拳。注重扶贫同扶志、扶智相结合。根据贫困人口意愿，积极开展职业技能培训，增强脱贫致富本领。开展移风易俗、孝善诚信专项活动，营造自强不息、勤劳致富社会风尚，激发贫困人口脱贫致富内生动力。加强贫困村基层组织建设，发挥贫困村基层党组织战斗堡垒作用。到2018年年底，力争6个贫困县全部达到摘帽标准，现行标准下农村贫困人口基本脱贫。

(二)坚决打好污染防治攻坚战。秉持绿水青山就是金山银山的理念，坚持节约优先、保护优先、自然恢复为主，统筹推进环境污染防治，确保环境质量持续好转，让“商丘蓝”成为常态，让商丘的水变清，让商丘的生态更美好。打赢蓝天保卫战，巩固和扩大现有治理成效，持续做好大气污染防治工作，严格各项防控措施，落实绿色化施工组织方式，确保空气优良天数保持在210天以上，PM10、PM2.5浓度完成省定目标任务。突出水环境治理，完成城区内河水系截污纳管、水面扩增、河岸生态绿化和生态湿地建设，中心城区黑臭水体彻底消除。对宁陵县清水河、睢县利民河、虞城县中心干渠等重污染水体实施综合治理，县城黑臭水体基本消除。健全沱河、包河、惠济河等流域水环境质量改善机制，确保5个国控出境断面水质稳定达标。严格水资源管理，完成三义寨引黄渠首移动泵站和闫谭提水工程建设，实施吴屯水库清淤扩容，加强饮用水水源地保护，积极支持配合做好引江济淮工程前期工作，促进我市水资源可持续利用。全面开展土壤污染防治，加强农业面源污染防治，分类实施农用地和建设用地环境管控，开展化肥、农药使用“零增长”行动，推进土地污染治理与修复，提升土壤质量。加强生态建设，完成黄河故道生态建设规划，巩固国家森林城市创建成果，继续实施生态廊道、农田防护林、平原沙荒造林、特色经济林等林业生态工程，全年计划造林10万亩以上。发挥黄河故道生态涵养功能，统筹推进森林、湿地、流域等生态系统修复工程，最终实现生产发展、生活美好、生态良好。

(三)加快先进制造业发展步伐。构建现代化产业体系，是量的扩张，更是质的提升。把加快先进制造业发展作为建设现代化产业体系的主攻方向，改造提升传统产业，培育战略性新兴产业，推动速度向质量转变、制造向创造转变、产品向品牌转变。按照《中国制造2025》的要求，制定企业准入标准，新上项目必须符合高端化、绿色化、智能化、融合化发展要求，提高制造业的核心竞争力和可持续发展能力。按照设备换芯、生产换线、机器换人的要求，整合资源设立产业发展基金和科技型中小微企业扶持基金，通过科技创新和“互联网+制造业”协同发展，对传统产业进行改造提升，推动存量实现提质发展，振兴商丘经济品牌，做优区域特色经济。按照节约集约土地的要求，制定产业项目投资强度和产出效益标准，强化用地规模管控，切实提高土地利用效率，建立健全节约集约用地长效机制。推进产业集聚区提质转型发展，突出抓好“四个五”重大产业项目，持续推动产业集聚区上规模上水平上层次，提高吸引力竞争力带动力。加快先进装备制造业园区发展，推动神火集团双零铝箔、海尔商丘工业园等一批大项目加快建设，实现增品种、创品牌、提品质的目标。夏邑县要确保印染产业园一期建成运营，以恒天永安智慧纺织项目为产业标准打造新型印染纺织产业基地。民权县加快北汽福田汽车装备和智能冷藏集装箱项目建设，带动提升制冷装备、冷链物流设备制造创新能力，推动产品智能化、自动化生产和升级换代。柘城县重点推进华晶金刚石二期项目，推动单晶向大颗粒宝石级产品发展，工业产品和钻石首饰并行提升。睢县重点发展安踏、特步、足力健等项目，进一步创建品牌、扩大规模，形成新的产能。虞城县加快推进电镀中心项目建设，确保科迪集团40万吨液态奶项目年内建成投产，在产业上创品牌、提效益。宁陵县加快推进LCD光电产业园项目建设，积极引进上下游及配套产业，促进产业转型升级。梁园区重点发展生物制药和医疗装备，形成集群集聚效应。睢阳区新材料工业园重点发展中建材光电产业园、国龙新材料等项目，培育新的竞争优势。力争柘城县、睢县、开发区晋升为二星级产业集聚区，虞城县、民权县争创全省“十强”产业集聚区。继续推进烟草转型升级，不断提高工作实效。促进酒业健康发展，重点推进张弓集团改制、九鼎葡萄酒公司重组、林河酒业帮扶和睢酒提质等工作，加快酒业结构调整和转型升级，重振商丘酒业辉煌。

(四)实施乡村振兴战略。推动农业农村优先发展，按照“产业兴旺、生态宜居、乡风文明、治理有效、生活富裕”的总要求，大力发展农村经济、社会、文化和生态文明，促进农村全面发展和繁荣。推进农业结构调整，启动粮食生产功能区和重要农产品保护区建设，新建高标准粮田50万亩，确保粮食产量稳定在120亿斤以上；大力发展生态、休闲、观光农业和都市设施农业，加快“三品一标”农产品认证，做优做强特色农业品牌，争创国家出口农产品质量安全示范区和国家生态原产地保护示范区，促进农业大市向优质农产品大市转变。扶持诚实人、福润等252个市级以上农业产业化龙头企业加快发展，积极推动土地流转，发展种粮大户、家庭农场、专业合作社等新型农业经营主体，推动农村一二三产融合发展，让农民参股，让农民受益，让农民的“钱袋子”鼓起来。全面提升农村基础设施，做好4个中小河流治理工程、引黄灌区续建配套与节水改造工程、38个贫困村农村饮水安全提升工程建设，建好、管好、护好、运营好农村公路，为加快推进农业农村现代化夯实重要基础。培养一支懂农业、爱农村、爱农民的“三农”工作队伍，建立农民工返乡创业激励机制，发挥其在乡村振兴中的重要作用，在发展农产品加工和乡村旅游、休闲农业、农村电商等方面施展才华，带富一方百姓，为农村基层组织建设增添力量。持续开展“美丽商丘·整洁村镇”活动，每个县选取30个试点，实现垃圾集中收集和污水集中处理，加大空心村和农村“双违”治理力度，改善农村人居环境。深入开展移风易俗，培育乡风文明，发挥乡俗民约、村规民约引领文明风尚的作用，真正让农民精神风貌好起来，让农村的文化生活丰富起来。

(五)积极发展现代服务业。服务业的繁荣发展是现代化的重要标志。以服务业“两区”为载体，注重统筹协调、同步提升、同步发展，促进新型工业化和现代服务业深度融合，形成相互促进、互为支撑的良好局面。2018年，确保全市服务业“两区”完成固定资产投资、主营业务收入和税收增长全部达到20%以上。中心城区重点围绕高铁枢纽商务区、日月湖现代服务业集聚区、古城文化旅游创新创意区三大区域，加快引进高端现代服务业，形成不以实现土地价值为目的，而以培育产业为目标的现代服务业集聚区。高铁枢纽商务区重点实施高铁叠城绿轴项目，加快高铁商城、中州世贸商城等项目建设，促进红星美凯龙爱琴海购物公园、汇豫国际商城、大悦城等项目尽快开工建设，着力打造城市交通综合体、生活休闲体验综合体、文化娱乐综合体等，营造和引领新的消费热点。日月湖现代服务业集聚区重点实施会展中心、建业总部港、金融创新大厦、互联网新经济大厦以及豫东规划设计中心、绿地五星级酒店群、人才公寓等项目，吸引总部经济、科技研发、金融、中介等高端服务业入驻，成为我市重要经济增长极。古城文化旅游创新创意区力争完成城墙修复、城湖恢复、城郭再现等重点项目，展现“三位一体”的古城独有风貌，加快中山大街和东西大街修复以及文庙、城隍庙等工程建设，发掘古城历史文化品牌，讲好商丘故事，展示家风文化、忠烈文化、廉洁文化等传统文化，在体验中丰富群众精神生活，促进旅游产业发展，形成以古城为龙头的全域旅游，叫响“游商丘古都城，读华夏文明史”旅游品牌，实现风貌修复、生产生活并重、并行推进的目标。用“互联网+流通”的方式，加快商丘农产品批发市场、中原车城等传统商贸流通企业和市场升级改造步伐。积极发展跨境电子商务“一中心三平台”建设，建设市跨境电商运营中心和邮政跨境物流产业园、中部(国际)跨境电商园、商丘进口商品保税园区，引入国际知名跨境电商入驻，加强与小红书、亚马逊、天猫国际等国内外知名电商企业战略合作，推进跨境物流转型升级，培育跨境电子商务企业20家以上。推进阿里巴巴(商丘)产业带提质增效，力争年底入驻企业突破1600家。以豫东综合物流产业集聚区为重点，加快推进传化公路港二期豫东配送分拨中心和物流大数据中心建设，中原佳海三期、亿丰国际三期等项目建成投入运营，推动豫东综合物流集聚区进入省多式联运试点。

(六)推进城乡统筹一体发展。加快综合交通枢纽建设。围绕构建高铁普铁“双十字加放射”枢纽网络，确保商合杭高铁商丘段项目顺利建设，开工建设商合杭新区站站房工程，力争京雄商高铁商丘段上半年开工建设。做好商丘航空港规划，开工建设商丘机场，推进永城、民权通用机场前期工作。加快沱浍河航运工程建设。保障阳新高速商丘段项目施工环境。加快“三环九放射”已开工5条路的建设进度，做好剩余4条路开工前的各项准备工作。依托市域快速路网体系，各县(区)加快谋划和推进域内路网建设，以交通道路建设为切入点，促进城乡统筹一体发展。加强以高铁枢纽站为核心辐射带动周边区域的快速路系统及中心城区快速路系统的规划设计研究，早日实现周边区域和中心城区均可快进快出高铁核心区的目标。打通胜利路、长江路、宋城路，向东与虞城相通，加快推进商虞一体化建设；延伸民主路、南京路、北海路，向西与机场、宁陵相接。中心城区按照“干道畅通、支路成网、四率管网优先”的原则，实施112条道路及管网配套建设项目，做好梁园路、睢阳大道、归德北路等“十纵十横”中的8条主干道工程，加快跨商合杭、京九高铁5个立交工程建设，长江路跨京九立交桥建成通车，实施北海路古城西出口等3个高速出入口互通式立交建设，推进道北、日月湖和先进装备制造园区周边次干道和支路网规划建设，完成50个路口渠化、80个公交港湾建设任务，强化道路和路口通行能力，建设停车位3万个以上，缓解停车难问题。通过综合交通枢纽建设，将交通优势转化为资源优势，释放区位、土地、人力资源等优势，建设大枢纽，带动大物流，构建大产业，推动大发展。推动重大基础设施统筹布局。在全市统筹布局垃圾无害化处理项目，统筹布局10千伏以上的输变电站工程及配网建设，统筹布局热源建设，2×35万千瓦热电联产项目辐射中心城区和虞城县城、产业集聚区及周边地区。

加快中心城区第二、第六污水处理厂建设，铺设污水管网70公里，实施中水回用工程，做到管网全覆盖、污水全处理、中水全利用。加快碧源水务30万吨水厂建设。实施环形主管网建设及长江路中继泵站和新建路、丰源换热站工程，完成15条道路供热管网新建改造工程。加大燃气管道铺设力度，铺设改造管网100公里，供气总量1.7亿立方米，新增民用燃气用户3万户。启动三大综合性公园和三大湿地公园建设，确保12个便民公园、30个街头游园建成开放，增加绿地166万平方米。实施人民公园改造提升工程，使其与高铁站北广场、绿轴共同形成良好的城市景观，带动周边发展。加强海绵城市试点建设，在新建公园、小区中，建成120个海绵体，实现雨水循环利用，有效补充城市水源。全面实施全国文明城市创建暨百城提质三年行动方案，全面启动国家卫生城市创建，对照各项创建指标，完善设施建设。按照繁华有序、畅通整洁的要求，持续做好城市精细化、规范化、数字化管理，大力开展“厕所革命”和城市“四治”，新建公厕30座，持续实施背街小巷全面提升工程，加强城市交通秩序管理，营造人人动手参与、共建美好家园的良好氛围，推动城市综合环境再上新台阶。促进房地产市场健康发展。加快2017年度安置房项目建设进度，中心城区在已开工5.2万套、729万平方米的基础上，今年再开工6.4万套、805万平方米；力争年内10900户、125.6万平方米建成入住，确保被征收群众早日迁入新居。3.2万套公租房年底前分配90%以上。2018年，全市计划实施棚户区改造项目41个，尽快启动改造和安置工作。坚持“房子是用来住的、不是用来炒的”定位，坚持居住小区功能配套完善的要求，适度加大住宅用地供应力度。大力推进租赁住房建设，规范租赁行为，适时推出限价商品房等措施，确保房地产市场平稳健康发展。加快示范区建设。坚持组团式开发、复合型建设的原则，推进开发区、物流集聚区、商务中心区“三大板块”建设，力争示范区固定资产投资、财政收入增速均保持在30%以上。努力将装备制造产业园建成以科技创新、先进制造、生态智慧为显著特征的先进制造业新城。市文化艺术中心建成投入使用，开工建设市图书馆、方志馆、市民中心和市委党校新校区等项目，完善城市功能。强化县城、镇区两大载体建设。坚持规划引领，加快永城区域副中心城市建设，做好县城城市设计，坚持新老并重，完善城区基础设施，促进产城融合，把县城打造成为承载产业和吸纳就业以及市政基础设施和公共服务设施向农村延伸的重要载体。加快推动重点镇建设，以综合交通建设带动乡镇发展，以产业发展带动就业增加，以医疗、教育和商业综合体建设完善功能。重点发展足力健幸福小镇、庄子文化小镇、石桥梨花小镇等为代表的特色小镇，使之成为农民就近转移就业的重要载体。依托历史文化遗存遗迹、自然禀赋、产业特色，梳理确定一批历史风貌特色村、自然风貌特色村、产业特色村等加快发展。

(七)全面深化改革扩大开放。进入新时代，改革开放的内涵更新、要求更高、任务更重。通过深化改革，倒逼政府职能转变，扫除阻碍发展的体制机制，提升政府治理能力和治理水平；通过扩大开放，倒逼企业转变生产组织方式，提升管理水平、产品品质和综合竞争力，为高质量发展夯实基础。推进全面深化改革。继续深化“放管服”改革，推进简政放权，做好行政许可事项的承接落实工作，对保留的行政审批事项、权责清单、公共服务事项进行动态调整，并向社会公布，接受群众监督。积极推行“互联网+政务服务”，打造“一网通办、一次办妥”政务服务平台，提高网上审批效率，让信息多跑路、群众少跑腿。深化商事制度改革，打造良好营商环境，进一步提升“三十五证合一”和证照全程电子化办理水平，建立事中事后监管清单，全面实施“双随机一公开”制度。清理废除妨碍公平竞争的各种障碍，进一步激发民营经济、中小微企业的活力和创造力，激发和保护企业家精神，构建亲清共融的政商关系。探索建立工业用地先租后让、租让结合、长期租赁等弹性办法，加快项目建设和产业发展；在适宜的区域探索按照被征用土地面积返还一定比例的建设用地，用于发展农村集体经济，增加被征地群众收入。推进国企改革攻坚，完成神火集团、路桥集团、市政工程总公司等改革改制工作。持续深化医药卫生体制改革，统筹推进盐业、价格等领域改革。持续深化农村改革，完善承包地“三权”分置制度，让农户的承包权稳下去、经营权活起来，提升集体经营性土地资本价值，有效增加农民财产性收入；统筹推进国有林场、农场、供销社、农村信用社等综合改革，为农业农村优先发展提供保障。积极实施开放带动战略。创新招商模式，探索建立财政资金、投融资公司、社会资本股权投资等多元化投资基金，建立产融结合的基金招商新模式，利用资本的催化和杠杆作用，吸引优质企业落户商丘，带动经济发展与产业结构升级。办好2018中国·商丘国际华商节，对外瞄准跨国公司和世界500强企业，对内紧盯国内行业领先龙头企业进行招商，充分发挥承接产业转移示范市的作用，引进一批具有资源整合力、产业带动力和区域影响力的项目。加快开放平台建设，积极争取河南自由贸易试验区异地共建区，依托商丘保税物流中心申建商丘综合保税区，推进铁路口岸查验区申建工作，与省电子口岸公司合作，大力拓展保税物流中心业务，扩充保税商品种类，建设进出口商品展销体验中心，争取更多优秀企业落户。

(八)坚持创新驱动发展。创新是引领发展的第一动力，是建设现代化经济体系的战略支撑。推动经济发展质量变革、效率变革、动力变革，提高全要素生产率，必须紧紧依靠创新驱动来实现。深入推进科技创新。整合全市科技资源，以一区多园的形式，争创商丘国家级高新区，睢阳产业集聚区争创省级高新区。加大财政科研经费投入，发挥财政资金的引导作用，带动更多的企业建立工程技术研究中心、重点实验室、院士工作站等研发机构，用科技创新引领发展。力争新增省级工程技术研究中心6家以上，新增高新技术企业10家，新增省级创新型科技团队1—2个。发挥商丘科学院作用，加快技术、人才引进，推动高等院校、科研院所在我市设立分支机构和成果转化基地。认真贯彻专利法，支持鼓励发明创造，切实保护知识产权。创新投融资机制。推动金融机构回归服务实体的本源，创新思路、降低门槛、简化手续，拓宽票据、合同、设备等抵押贷款渠道，提高服务实体经济能力。研究出台支持企业主板上市的激励政策。加快政府融资平台市场化改制，形成集“借、用、建、管、还”为一体、完全独立、自主经营的市场主体，通过政府购买服务，使之成为政府特许经营的持有者、公共服务的经营者、公共产品的供给者和重点产业的扶持者，为城市建设和产业发展提供坚强保障。培育发展新经济。以提高供给质量、优化供给结构为主攻方向，推动互联网、物联网、人工智能和传统产业深度融合，改革先进制造业、现代服务业发展组织方式，提升产业发展水平。加快商丘大数据中心和颐高“双创”园建设，拓展数字消费、电子商务、共享经济、智慧经济等新业态新模式，为高质量发展提供强大动力。创新引才聚才机制。实施更加积极的激励政策，通过培育中高端产业引进中高端人才，促进人才队伍向产业集聚、创新创业要素向人才倾斜，加快建设科技创新领军人才和高技能人才队伍，建设产业高地、创新高地。着力打造技术服务、成果交易、创新创业融资服务平台，在住房、就业、薪酬待遇、科研成果转化等方面制定出台支持政策，吸引专科生、本科生以及更高学历的人才来商丘就业创业，为推动创新发展提供强有力的智力支撑。

(九)加快公共产品供给。坚持以人民为中心的发展思想，在经济发展的同时，不断增加财政支出，办好事关人民群众切身利益的民生实事，努力在“幼有所育、学有所教、劳有所得、病有所医、老有所养、住有所居、弱有所扶”以及美丽商丘建设等方面取得新进展，不断满足群众对美好生活的需要。提高就业质量。继续实施全民技能振兴计划，开展职业技能培训，鼓励退役军人和科技人员等创业，以创业带动就业，年内新增城镇就业5.5万人以上。发挥创业孵化园区、高校科研资源和研发优势，为高校毕业生和在校大学生提供创业场地、补贴和指导服务。优先发展教育事业。做好中小学空间布局规划，完成“全面改薄”任务，推动义务教育均衡发展。开工建设市回民中学、市第一实验小学新校区，力争一中、一高新校区秋季招生。中心城区新建35所中小学，其中梁园区9所、睢阳区16所、示范区10所；加快寄宿制学校建设，确保县城建有2—3所寄宿制高中，所有乡镇建有1—2所寄宿制中学、2—3所寄宿制小学；中心村建有寄宿功能的小学，利用农村闲置校舍建设一批幼儿园。引导和规范民办教育发展，满足群众不同层次教育需求。支持高校发展，推动学科设置和产业集聚区主导产业用工需求相结合，把专业办在企业，把教室搬进车间，把车间建在学校，培养高技能劳动大军。加强师德师风建设，培养高素质教师队伍。建设商丘文化高地。深入挖掘商文化、火文化、汉梁文化等文化资源，保护利用好四平调、麒麟舞、泥人李等非物质文化遗产，引导发展民间工艺，培育集创意研发、生产销售、文化体验为一体的传统民间工艺品集散区，建设豫东民间小吃精品区。继续推进文化下乡、农家书屋建设、农村电影放映等文化惠民工程。大力推进全民阅读，建设书香商丘。编制好大运河遗址后续保护规划，启动宋国故城考古发掘工作，逐步建立隋唐大运河考古博物馆、古都城博物馆、火文化、民俗文化等具有商丘特色的博物馆体系，打造文化品牌，擦亮文化符号，增强文化自信。健全社会保障体系。继续推动全民参保计划，全面实施机关事业单位养老保险制度。深化医保支付方式改革，持续推行省内异地就医即时结算和跨省异地就医直接结算，全面实施先诊疗后付费结算机制。完善社会救助体系，全市建设100个社区服务中心，改造提升100所敬老院，建成1000所农村留守儿童之家。推进健康商丘建设。推进城市公立医院综合改革，完善社区卫生服务网络，加快医联体建设，积极推行分级诊疗和家庭医生签约服务。加快推进市中医院新院区、市传染病医院西院区等项目建设，推动市第一人民医院心血管、肿瘤治疗中心和神经专科医院早日开工建设。推动中医药事业振兴发展。积极应对人口老龄化，大力推进健康养老产业转型发展，在健康养老示范项目建设、医养深度融合取得新成效。积极开展丰富多彩、形式多样的全民健身运动，促进运动和健康同步发展。

(十)防范化解重大风险。在推进经济社会发展过程中，我们有更多的挑战需要应对、更多的风险需要防范、更多的困难需要克服，我们将增强忧患意识、风险意识、底线思维，努力争取把风险化解在源头，牢牢守住不发生系统性风险的底线。防范化解政府性债务风险。严格控制债务规模，规范政府举债行为，严禁违规举债，使债务规模与经济发展水平、财力承受能力相适应。加强源头管理，将政府性债务分门别类纳入政府预算统一管理，确保风险可控。做好非法集资和问题楼盘化解工作。加大防范非法集资宣传力度。切实维护群众的合法权益，完善处非工作长效机制，加强对担保公司、小额贷款公司、投资类企业、典当行等机构的监管，防止民间借贷风险向银行业体系传导，确保不发生区域性、系统性金融风险。总结问题楼盘处置经验，完善处置机制，拓宽问题楼盘融资渠道，引进有实力的大型企业，对问题楼盘进行投融资一体化建设，力争问题楼盘早日得到妥善解决。创新社会治理。推进共建共治共享，做好新形势下群众工作，认真落实“三·五”基层工作日制度，完善矛盾纠纷多元化解机制。加强法律援助工作，让群众在每一个司法案件中都能感受到公平正义。加强社区建设，推进老旧小区和无主管楼院的提升改造工作。落实政治待遇、工资待遇、基本养老、医疗等保障，提高社区工作者工作积极性。做好第九届村委会和第六届居委会换届选举工作。推进平安商丘建设。建立立体化信息化社会治安防控体系，依法打击违法犯罪活动，不断提升群众安全感和满意度。健全食品药品监管体系，积极开展食品安全市县创建，让食品药品更安全，让老百姓更放心。加强道路交通、建筑工地、消防等重点领域安全隐患排查，落实安全生产责任制，健全事故应急机制，坚决防范各类生产安全事故发生，确保实现“三下降一杜绝”的目标。

继续推进全民国防教育、国防动员和国防后备力量及人民防空建设，支持驻商部队建设，积极开展双拥工作，争创全国双拥模范城“八连冠”，推进军民融合发展。加强精神文明建设，进一步叫响“商丘好人”品牌。支持工会、共青团、妇联等人民团体发挥桥梁纽带作用。做好民族宗教工作。统筹做好统计、外事侨务、机关事务、史志、档案、防震减灾、气象、邮政、通信和对口援疆、区域经济合作等工作。

三、以党风带政风全面加强政府自身建设

各位代表！党的十九大提出了新时代党的建设的总体要求和重大任务，是我们做好一切工作的根本遵循。全市政府系统将以习近平新时代中国特色社会主义思想为指引，践行新时代党的建设总要求，加快推进政府各项工作，努力建设人民满意的政府。

(一)提升政治站位。深入贯彻党的十九大精神，用习近平新时代中国特色社会主义思想武装头脑，牢固树立“四个意识”，增强“四个自信”，严守政治纪律和政治规矩，坚决维护以习近平同志为核心的党中央权威，在政治立场、政治方向、政治原则、政治道路上同以习近平同志为核心的党中央保持高度一致。坚定跟着党中央令旗走，坚定执行党的路线，让党的各项方针政策在商丘落地生根、开花结果。持续推进“两学一做”学习教育常态化制度化，认真开展“不忘初心、牢记使命”主题教育。

(二)增强工作本领。把法治思维贯穿政府工作全过程，自觉带头学法、带头用法、带头守法，认真办理人大代表建议和政协委员提案，自觉接受人大、政协和社会监督。完善政府法律顾问制度，坚持科学决策、民主决策、依法决策，落实行政执法责任制，深入推进服务型执法。始终保持锐意进取的精神风貌，不断更新知识层次和思维方式，善于处理各种复杂矛盾，推动政府管理创新、制度创新、工作创新，确保政府工作更富创造性。

(三)强化责任担当。进一步弘扬“红船精神”、井冈山精神、长征精神、延安精神和焦裕禄精神，坚持开拓进取，强化责任担当，勇于改革创新。紧紧围绕重大工作部署和重点任务，加强调查研究，凝心聚力，真抓实干，确保取得实实在在的效果。按照“马上办抓落实”和“六位一体”工作推进机制的要求，认真解决发展中的新矛盾、新问题，努力在落实新发展理念上有新作为、在深化改革上有新突破、在改善民生上有新成效。

(四)做到廉洁从政。全面加强政府系统作风建设，自觉践行“三严三实”要求，坚决落实中央八项规定精神，驰而不息纠正“四风”问题。严格履行党风廉政建设责任制，坚定不移推进反腐败斗争，保证干部清正、政府清廉、政治清明。健全行政权力约束机制，持续强化对政府投资、工程建设招投标等重点领域、关键环节的监督，从源头上预防腐败，以清廉的政风赢得群众的信任和支持。

各位代表！新时代开启新征程，新征程呼唤新作为。让我们更加紧密团结在以习近平同志为核心的党中央周围，在省委省政府和市委的坚强领导下，高举习近平新时代中国特色社会主义思想伟大旗帜，不忘初心，牢记使命，砥砺奋进，锐意进取，为决胜全面建成小康社会、开启商丘现代化建设新征程而不懈奋斗！
